# Molecular characterization of human ABHD2 as TAG lipase and ester hydrolase

**DOI:** 10.1042/BSR20160033

**Published:** 2016-07-15

**Authors:** Naresh Kumar M., Thunuguntla V.B.S.C., Veeramachaneni G.K., Chandra Sekhar B., Swapna Guntupalli, Bondili J.S.

**Affiliations:** * Department of Biotechnology, K L University, Green fields, Vaddeswaram, Guntur District, Andhra Pradesh 522 502, India

**Keywords:** α β hydrolase domain, ester hydrolase, lipid metabolism, triacylglycerol (TAG) lipase

## Abstract

Alterations in lipid metabolism have been progressively documented as a characteristic property of cancer cells. Though, human *ABHD2* gene was found to be highly expressed in breast and lung cancers, its biochemical functionality is yet uncharacterized. In the present study we report, human ABHD2 as triacylglycerol (TAG) lipase along with ester hydrolysing capacity. Sequence analysis of ABHD2 revealed the presence of conserved motifs G^205^XS^207^XG^209^ and H^120^XXXXD^125^. Phylogenetic analysis showed homology to known lipases, *Drosophila melanogaster* CG3488. To evaluate the biochemical role, recombinant *ABHD2* was expressed in *Saccharomyces cerevisiae* using pYES2/CT vector and His-tag purified protein showed TAG lipase activity. Ester hydrolase activity was confirmed with pNP acetate, butyrate and palmitate substrates respectively. Further, the ABHD2 homology model was built and the modelled protein was analysed based on the RMSD and root mean square fluctuation (RMSF) of the 100 ns simulation trajectory. Docking the acetate, butyrate and palmitate ligands with the model confirmed covalent binding of ligands with the Ser^207^ of the GXSXG motif. The model was validated with a mutant ABHD2 developed with alanine in place of Ser^207^ and the docking studies revealed loss of interaction between selected ligands and the mutant protein active site. Based on the above results, human ABHD2 was identified as a novel TAG lipase and ester hydrolase.

## INTRODUCTION

Lipases in addition to being essential enzymes necessary for the supply of energy also play a major role in lipid signalling and metabolism. Aberrations in lipases were found in many human diseases, including cancer, making it imperative to understand these enzymes. Recently, the mammalian α β hydrolase domain (ABHD) containing proteins have emerged as novel potential regulators of lipid metabolism and in signal transduction [1]. The human ABHD family contains 21 proteins and is part of a superfamily possessing an α β hydrolase fold [1,2]. Hydrolase activity of ABHD is attributed to the catalytic triad composed of serine-acid-histidine residues located in loop regions. The corresponding motif found in most of the human ABHD family members was GXSXG. The acid residue of the catalytic triad can either be a glutamate or aspartate, usually located after strand β7 [3]. Interestingly, the majority of the human ABHD proteins also possess another conserved motif HXXXXD where X is any amino acid residue and the motif was attributed to acyltransferase activity [4].

Some of the human ABHD proteins were well characterized as lipases, including ABHD3 which selectively cleaved medium-chain and oxidatively-truncated phospholipids. ABHD4 was described as lyso-*N*-acyl phosphatidylethanolamine lipase [5–7]. Human ABHD5, also known as Comparative Gene Identification 58 was found to be the causative gene of human Chanarin Dorfman Syndrome, also known as the neutral lipid storage disease (NLSD) [8,9]. ABHD6 and ABHD12 were identified as 2-arachidonylglycerol (AG) hydrolase which plays a key role in neurotransmission [10–14]. Later, *ABHD12* was also found to be linked with an autosomal recessive genetic disorder called Usher Syndrome 3 [15]. Human lymphocyte antigen B-associated transcript 5 (BAT5), also known as ABHD16A was shown hydrolysing medium and long-chain unsaturated monoacylglycerols (MAGs).

Human *ABHD*2, previously known as lung α/β hydrolase 2 (*LABH2*), is one among the α β hydrolase superfamily [16]. Reduction in the number of alveolar type II cells and unusual accumulation of macrophages in the lungs was seen in aged mice by global deletion of *ABHD*2 [17]. In addition to its role in the lung, *ABHD*2 plays a significant role in macrophage infiltration to atherosclerotic lesions [18]. Collectively, *ABHD*2 seems to play an important role in chronic diseases, i.e. atherosclerosis and emphysema involving monocyte/macrophage recruitment. However, this putative lipase has not been studied in any detail and there are no experimental data to confirm the functionality, evolutionary relationship, substrate specificity and the role of this protein in lipid breakdown. In the present study, we report the triacylglycerol (TAG) lipase and ester hydrolase activities of human α β hydrolase2 (ABHD2).

## EXPERIMENTAL

### Strains and growth conditions

Yeast strains used in the present study are *Saccharomyces cerevisiae* BY4741 (WT), *ABHD*2 overexpressed in WT (OE) and only vector pYES2/CT cloned in WT (v). WT cells were grown either in YPD medium containing 1% yeast extract, 2% peptone and 2% dextrose weight/volume (w/v) or synthetic minimal medium (SC+Ura) containing 0.67% yeast nitrogen base (YNB), supplemented with the complete supplement mixture 0.192% appropriate amino acids without uracil, 2% dextrose and 0.015% uracil (w/v). Recombinant yeast strains OE and V were cultured in synthetic minimal medium without uracil (SC-Ura) containing 0.67% YNB, supplemented with the complete supplement mixture 0.19% appropriate amino acids without uracil and 2% dextrose. Induction was done in SC-Ura media with 2% raffinose and 3**×**YP medium with 6% galactose [19]. All cells were cultured in liquid media at 30°C with shaking at 180 rpm.

### Phylogenetic analysis

Molecular phylogenetic analysis was performed with the 21 human ABHD family proteins retrieved from NCBI along with orthologous sequences of mouse, rat, *Drosophila melanogaster* and *Arabidopsis thaliana*. The tree was developed by maximum likelihood method using MEGA (version 6.0) [20]. Bootstrap values were determined from 1000 trials and the phylogenetic tree with the highest log likelihood is shown. The tree was drawn to scale and the analysis involved a total of 25 protein sequences.

### Cloning and expression of recombinant ABHD2

*ABHD2* was cloned into pYES2/CT vector and transformed into DH5α cells. Only vector and pYES2/CT along with the construct were further transformed into WT individually by using the Frozen-EZ Yeast Transformation kit (Zymo Research) following the manufacturer protocol. Expression of the recombinant *ABHD2* in WT was performed as per Gelperin et al. [19] and purified by Ni-NTA agarose (Qiagen) column.

### Esterase activity using *p*-nitrophenyl esters as substrates

Esterase activity was carried out with *p*-nitrophenyl acetate (pNPA), *p*-nitrophenyl butyrate (pNPB) as mentioned by Ploier et al. [21]. Similarly, for *p*-nitrophenyl palmitate (pNPP) substrate assay was conducted as per Kanwar et al. [22]. Different concentrations of substrates (2–20 mM) were considered for analysis of enzyme kinetics with all the three substrates studied. The purified recombinant ABHD2 enzyme of 3.34 μg was used for esterase activity against pNPA, pNPB and 33.40 μg with pNPP substrates respectively. Hydrolytic activity was checked at different pH 4.5, 5.5 (sodium acetate buffer), 6.5, 7.5 (sodium phosphate buffer), 8.5 and 9.5 (Tris/HCl buffer) with all the substrates. The esterase activity was also monitored at different temperatures, including 4, 30, 45, 60, 75 and 90°C. All the assays were performed in triplicates and mean values were recorded. Michaelis–Menten kinetics was analysed using Graph Pad Prism version 5*.*

### TAG lipase assay

TAG lipase activity was assayed using Lipase activity assay kit (K722-100; Biovision) following the manufacturer protocol. In brief, lipase hydrolyses a triacylglycerol substrate to form glycerol which is measured enzymatically at 570 nm, by monitoring a linked change in the OxiRed probe absorbance [23]. The lipase assay was carried out with 3.34 μg of purified enzyme at pH 8.5 and incubated at 37°C.

### Homology modelling

The query ABHD2 sequence was retracted from the Uniprot resource (P08910) [24]. Homology modelling was carried using the Prime module of the Schrödinger suite [25]. The template for the model building was searched using the blast search bundled within the software. The secondary structure was predicted using the SSpro module. Following the ClustalW protocol, both the target and the template were aligned and finally the model was built omitting the inbuilt ligands if any. The stereo chemical quality of the model was assessed by using PROCHECK [26].

### Molecular dynamic simulation studies

The model was further refined using the Desmond molecular dynamic simulations (MDS) [27]. With system builder of the Desmond module, the model was incorporated in an orthorhombic periodic boundary box and was solvated using the SPC water model. This was neutralized with 4Na^+^ ions based on the total charge of the model and to this system 0.15 M salt was added. This was simulated using the dynamic simulation step of the Desmond module. The simulations were carried using NPT ensemble, at 300 K temperature, one atmospheric pressure and finally the entire system was relaxed using the default protocol. This system was carried for 100 ns simulation time period and the trajectory obtained from this simulation run was analysed.

### Ligand preparation

The structures for the molecules were retrieved from Pubchem database [28] and were prepared using LigPrep tool [29] of Schrödinger suite with OPLS_2005 as a force field. The ionization states of these molecules were generated at pH 7.0±2.0 using Epik module and 32 possible stereoisomers generation per ligand was selected.

### Docking studies

The refined model obtained from the simulation studies was further prepared using the protein preparation wizard [30] by addition of hydrogens and bond orders assignment. The model was then optimized and minimized using force field OPLS_2005 with RMSD of 0.30 Å (1 Å=0.1 nm). A grid box was generated around the important amino acids reported previously using the receptor grid generation protocol of the Glide module [31]. The prepared protein and molecules were docked covalently using the covalent docking protocol of the glide module.

### *In silico* mutational analysis

The model was point mutated with Alanine replacing the Ser^207^ of the catalytic traid and the mutated model was docked with selected pNP substrates under the similar experimental conditions opted above.

## RESULTS AND DISCUSSION

Variations in lipid metabolism have been increasingly listed as one of the characteristic features of cancer cells. DNA-microarray data from the ONCOMINE database indicated differential expression of lipases and other proteins related to the α β hydrolase family in various tumours [32]. In addition, the multiple data sets obtained from breast cancer in comparison with normal cells also indicated high expression of human *ABHD2* gene in breast and lung cancers [33–35]. This prompted us to check the functionality of human ABHD2 protein. *In silico* sequence analysis highlighted only the putative functional role but the substrate specificities of the enzyme are not yet clearly depicted. Present study identified human ABHD2 as both TAG lipase and ester hydrolase based on experimental data.

### Domain structure

**Figure 1:**
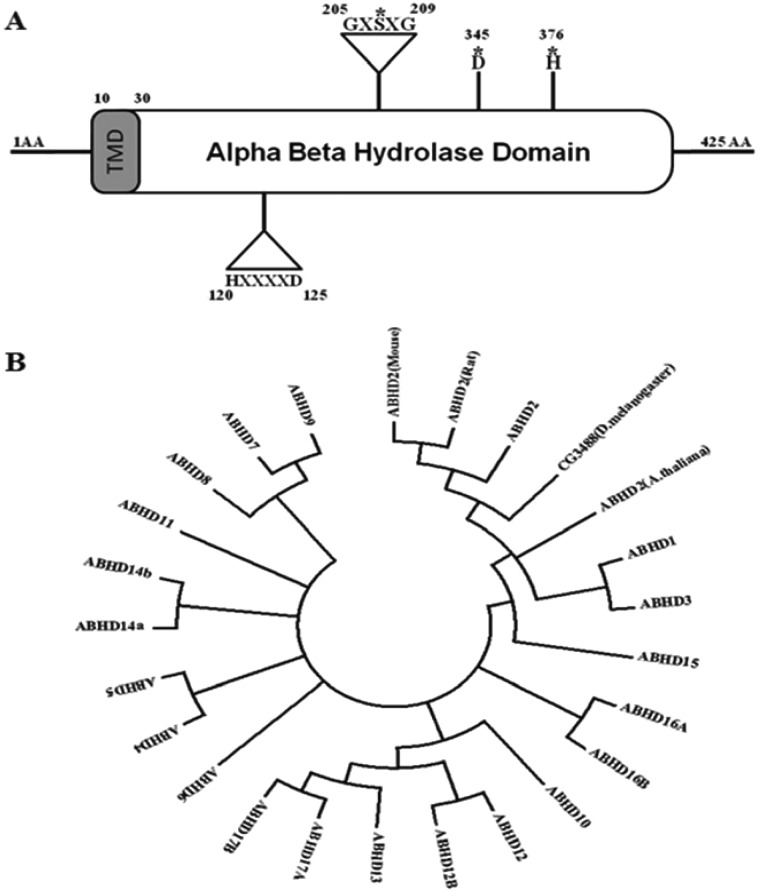
Domain structure and phylogenetic analysis of Human ABHD2 (**A**) Dual signature motifs of ABHD2: The positions of the conserved GXSXG lipase and HXXXXD acyltransferase motifs in ABHD2 are indicated by triangles with the respective amino acid residue positions and the key amino acids involved in α/β hydrolase domain catalytic triad are represented with (*) mark. (**B**) Phylogenetic analysis using the maximum likelihood method: Molecular phylogenetic analysis was performed with the sequences of Abhd2 (mouse) gi|33333856, Abhd2 (rat) gi|157820401, *CG3488*/Abhd2 (*D. melanogaster*) gi|27923956, Abhd2 (*A. thaliana)* gi|15240586 and all 21 human (h) ABHD family proteins hAbhd1 gi|308153404, **hAbhd2** gi|23397661, hAbhd3 gi|134035377, hAbhd4 gi|74762601, hAbhd5 gi|31542303, hAbhd6 gi|189027141, hAbhd7 gi|134035378, hAbhd8 gi|74732007, hAbhd9 gi|92095996, hAbhd10 gi|8923001, hAbhd11 gi|74751292, hAbhd12 gi|109689718, hAbhd12B gi|50401854, hAbhd13 gi|74749881, hAbhd14A gi|143955271, hAbhd14B gi|33991637, hAbhd15 gi|308153403, hAbhd16A gi|23813746, hAbhd16B gi|23813959, hAbhd17A gi|194306562, hAbhd17B gi|74746845.

*In silico* sequence analysis revealed human ABHD2 (P08910), as a protein of 425 amino acids containing an α/β hydrolase domain ranging from 1 to 425 amino acids and belonging to α/β hydrolase superfamily. The conserved GXSXG sequence motif is found between 205 and 209 amino acids (Figure 1A) which is the general substrate binding site of lipase and ester hydrolase enzymes. Active sites identified are serine (S^207^), aspartic acid (D^345^) and histidine (H^376^) (Figure 1A). Another conserved motif HXXXXD sequence is found in the N-terminal region, ranging from 120 to 125 amino acids (Figure 1A). A trans-membrane region is also found in the protein sequence at the N-terminal spanning 10–30 amino acids (L^10^PAVFDGVKLAAVAAVLYVIV^30^) as predicted by DAS-TM filter server and also identified as a type II membrane protein by Innovagen peptide property calculator software with poor water solubility hydropathy plot.

### Sequence homology and phylogenetic analysis

Sequence comparison of human *ABHD2* (gi: 23397661), with annotated databases revealed sequence similarity with *D. melanogaster CG3488*, mouse ABHD2, rat ABHD2, *A. thaliana* ABHD2 and human ABHD family members. The phylogenetic tree diverged into three groups (Figure 1B) containing, Group 1 with *D. melanogaster* CG3488, ABHD2 of mouse, rat, and *A. thaliana* along with human ABHD 1, 2, 3 and 15*.* Human ABHD10, 12 and 12B, 13 along with 17A and B formed the second Group. The rest of the human ABHD sequences grouped together including ABHD 4, 5, 6, 7, 8, 9, 11, 14A, 14B, 16A and B. This clearly highlights the homology of ABHD2 with known TAG lipase of *D. melanogaster* CG3488 [36]. Plant orthologue (*A. thaliana*) has been yet uncharacterized.

### TAG lipase activity

To determine the hydrolytic activity, human *ABHD2* gene was overexpressed in *S. cerevisiae* WT cells and the recombinant protein was purified to perform enzymatic assays. For this purpose, full-length protein of 51.7 kDa was expressed with a C-terminal 6 ×His-tag using pYES2/CT vector and was successfully purified from whole cell extracts by Ni-NTA column. This affinity purified recombinant hABHD2 protein fraction showed TAG lipase activity of 1.14±0.11 μmol/s·mg of protein against controls.

### Esterase assay confirms hydrolytic activity

**Figure 2:**
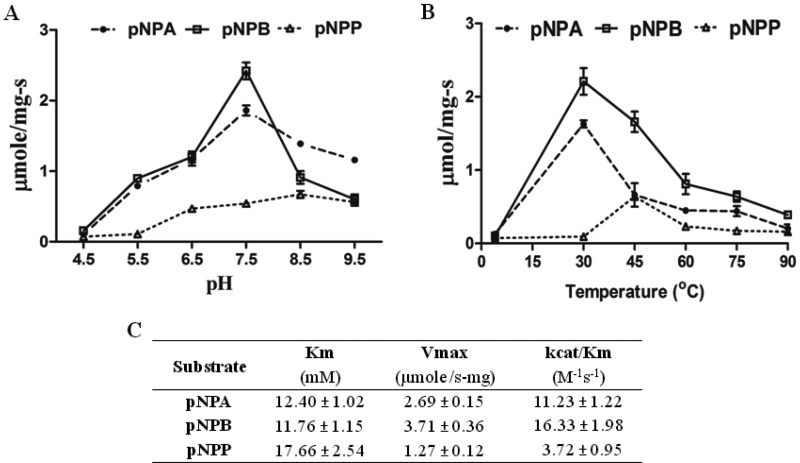
Ester hydrolase activity of ABHD2: kinetics, pH and temperature optima with pNP substrates (**A**) Highlights the optimum pH value showing maximum enzyme activity. (**B**) Represents the ABHD2 activity at different temperatures. (**C**) Purified ABHD2 enzyme of 0.67±0.06 mg/ml concentration was used for the analysis of esterase activity using pNPA, pNPB and pNPP as substrates. Data were analysed using graph pad prism 5. Data are mean values from three independent experiments with respective S.D. values.

The purified recombinant ABHD2 enzyme was used for esterase activity against pNPA, pNPB and pNPP as substrates. Hydrolytic activity was screened at different pH and was found to be optimum at pH 8.5 for pNPP and at pH 7.5 for both pNPA and pNPB substrates respectively (Figure 2A). The esterase activity was also monitored at different temperatures and was found optimum at 45°C for pNPP (Figure 2B) whereas pNPA and pNPB substrates showed optimum activity at 30°C.

ABHD2 cleaved, pNPA with a *K*_m_ of 12.40±1.02 mM, *V*_max_ of 2.69±0.15 μmol/s·mg and *k*_cat_/*K*_m_ of 11.23±1.22 M^−1^·s^−1^, pNPB with a *K*_m_ of 11.76±1.15 mM, *V*_max_ of 3.71±0.36 μmol/s·mg with *k*_cat_/*K*_m_ of 16.33±1.98 M^−1^·s^−1^ and pNPP with a *K*_m_ of 17.66±2.54 mM, *V*_max_ of 1.27±0.12 μmol/s·mg and *k*_cat_/*K*_m_ of 3.72±0.95 M^−1^·s^−1^ (Figure 2C). pNPP is not very water soluble and likely affects the apparent *V*_max_, but it is found to be a substrate when compared with control data. Control assay with only pYES2/CT vector alone overexpressed and purified fractions showed no activity. All together, the present study highlights the TAG lipase activity of ABHD2 along with both long and short chain esterase activities against pNP palmitate, butyrate and acetate substrates respectively.

### Homology modelling

**Figure 3:**
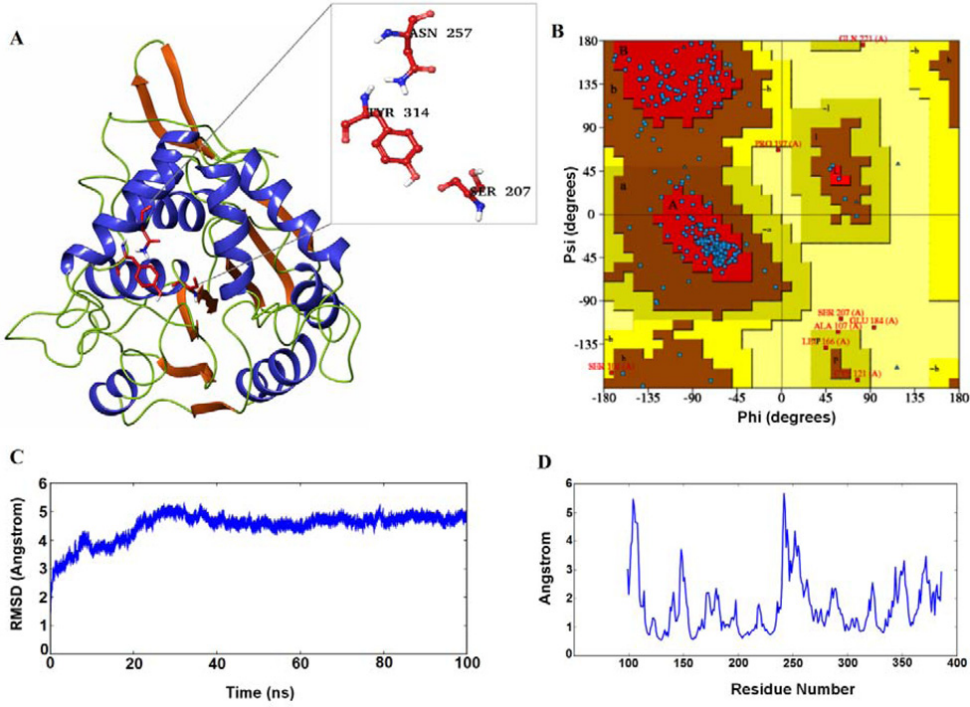
Homology modelling and MDS (**A**) The predicted 3D model of ABHD2 after clustering; (**B**) Ramachandran plot analysis of the built model; (**C**) RMSD graph of the model obtained after the 100 ns simulation run; (**D**) RMSF of the amino acids plotted using the trajectories obtained by the 100 ns MDS.

A 3D model was required to perform the binding studies, for which we opted prime modelling tool of the Schrodinger suite. Based on the BLAST results, 1BRO of *Streptomyces aureofaciens* with a resolution of 2.05 Å was chosen as a template to build the structure. This protein showed 24% identity and 46% positives against our query sequence, which was the top and best among the BLAST hits obtained. The background for considering this particular PDB file apart from the above points was it contains α/β hydrolase domain and also the important catalytic triad. The model was built (Figure 3A) based on this template and was further analysed using Ramachandran plot. 81.5% of residues were reported to be in the most favoured regions, 15.8% residues in additionally allowed regions (Figure 3B), 1.5% in generously allowed regions and remaining 1.2% only in the disallowed regions of the Ramachandran plot. Based on this result, the model was predicted as the best one and was further refined using the MDS studies.

The model was further refined and analysed based on the RMSD and RMSF of the trajectory obtained after the 100 ns simulation run. Deviations in the model were reported between 1.5 and 5.2 Å during the simulation run period. During the initial run time of simulations, the model showed huge deviations from 1.5 to 5.2 Å up to 30 ns (Figure 3C). From there, the deviations showed small declination from 5.2 Å to below 5.0 Å and further very small inclinations and declinations were reported with a steady state approximately 5.0 Å. The deviations in the initial stages were mainly due to the presence of a number of loops in the protein structure. The movement of amino acids of the modelled protein was analysed using the RMSF plot (Figure 3D). High fluctuations were reported above 5.0 Å between 100–110 and 240–250 amino acids of the model. Other residues between 140–155 and 250–265 displayed fluctuations within 4.0–5.0 Å. The remaining amino acids were reported below 3.0 Å. The majority of the amino acids which were fluctuating rapidly during the simulations run time were present in the end loop region and attained stability by the end of dynamics time period. The fluctuation range in the modelled protein was reported approximately 0.6–5.8 Å.

### Ligand docking studies

The frames obtained from the trajectory were divided into clusters based on their energies. The cluster, which was displaying the minimum energy was continued for the binding studies. During the homology modelling, the model was built without using the template crystal ligand data. Hence, based on the previous literature, catalytic residues of the active pocket were predicted and through the receptor grid generation protocol of the glide module, the selected pocket was fixed. Three ligands were docked into the fixed active pocket of the model following the covalent docking protocol. Based on G-Scores, the three molecules binding modes were analysed.

**Figure 4:**
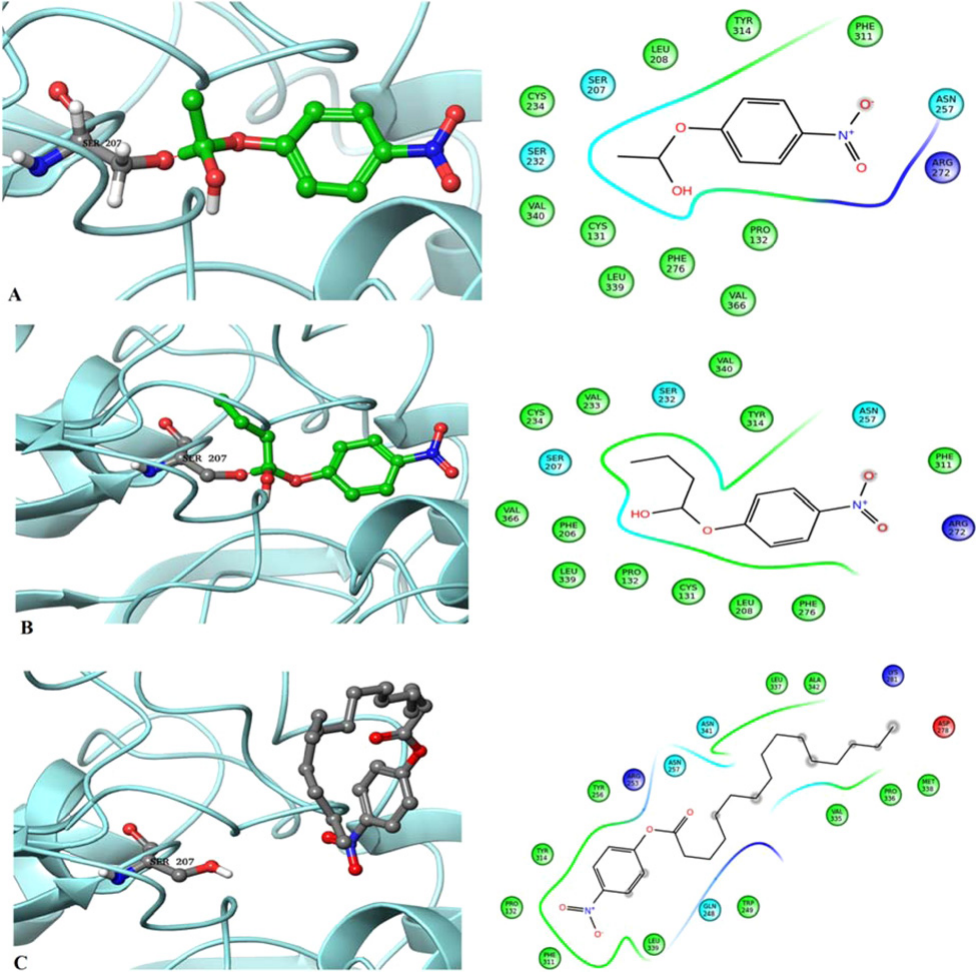
ABHD2 docking interactions with pNP substrates Covalent bond formation between the ABHD2 model with (**A**) pNPA; (**B**) pNPB and (**C**) pNPP highlighting the interactions with specific amino acids.

Three molecules pNPA, pNPB and pNPP were initially prepared using the LigPrep. The prepared molecules were covalently docked into the receptor active pocket i.e. catalytic triad. Both acetate and butyrate molecules produced covalent bond with the serine of the catalytic triad which was the key residue in performing the hydrolase activity. The developed ABHD2 model and the acetate ligand shared covalent bond between the oxygen atom of serine and carbon atom of acetate with a G-score of −3.21 (Figure 4A). The ABHD2–butyrate complex also produced covalent bond sharing electrons between the oxygen and carbon atoms as depicted in Figure 4(B). This complex showed G-score of −3.63 which was slightly higher than the ABHD2–acetate complex. This is in agreement with the *in vitro* activity observed with the same substrates. Whereas in the ABHD2–palmitate complex, instead of the covalent bond, one hydrogen bond and one pi–pi stacking were observed (Figure 4C). There was no interaction between palmitate and any of the important residues of the catalytic triad because the fitting of the ligand was away from the predicted pocket. The hydrogen bond was formed with the residue Asn^257^ and the stacking was with Tyr^314^ with a G-score of −3.50.

Further, the model was cross validated with point mutated ABHD2. The important residue of the active site Ser^207^ was replaced with Alanine. The mutated model showed no interactions with any of the chosen substrates when covalently docked (Supplementary Figure S1). This result highlights the role of Ser^207^ in forming covalent bonding with the substrates and its importance in the activity of lipases with conserved GXSXG motif. These observations correlate with the *in vitro* mutational studies of lipases, MGAT of *Arachis hypogaea* [37] Cvt17 [38] Lpl1 [39], ROG1 [40] of *S. cerevisiae* which showed no or marginal activity when serine of GXSXG motif was mutated with alanine.

## CONCLUSION

In conclusion, the human ABHD2 protein sequence shows an α/β hydrolase domain with conserved GXSXG lipase motif. To study the role of this conserved GXSXG domain, *ABHD2* was cloned, overexpressed and the recombinant protein was used for *in vitro* enzyme assays. The results obtained with His-tag purified recombinant ABHD2 protein clearly displayed both TAG lipase and ester hydrolase activities. This functionality justifies the increase in expression of human *ABHD2* gene in breast and lung cancers, supporting the necessary energy by the breakdown of lipids for the accelerated proliferation of cancer cells. Thereby, it could also serve as a selective and potential target for combinatorial cancer chemotherapy.

## Abbreviations

ABHD: α β hydrolase domain
MDS: molecular dynamic simulation
pNP: *p*-nitrophenyl
pNPB: pNP butyrate
pNPP: pNP palmitate
pNPA: pNP acetate
RMSF: root mean square fluctuation
TAG: triacylglycerol
w/v: weight/volume
WT: wild type
YNB: yeast nitrogen base
